# Redox Response of Reduced Graphene Oxide-Modified Glassy Carbon Electrodes to Hydrogen Peroxide and Hydrazine

**DOI:** 10.3390/ma6051840

**Published:** 2013-05-07

**Authors:** Shigehiro Takahashi, Naoyuki Abiko, Jun-ichi Anzai

**Affiliations:** Graduate School of Pharmaceutical Sciences, Tohoku University, Aramaki, Aoba-ku, Sendai 980-8578, Japan; E-Mails: t-shigehiro@m.tohoku.ac.jp (S.T.); n.abiko@nippon-soda.co.jp (N.A.)

**Keywords:** graphene, electrodeposition, GC electrode, hydrazine, hydrogen peroxide

## Abstract

The surface of a glassy carbon (GC) electrode was modified with reduced graphene oxide (rGO) to evaluate the electrochemical response of the modified GC electrodes to hydrogen peroxide (H_2_O_2_) and hydrazine. The electrode potential of the GC electrode was repeatedly scanned from −1.5 to 0.6 V in an aqueous dispersion of graphene oxide (GO) to deposit rGO on the surface of the GC electrode. The surface morphology of the modified GC electrode was characterized by scanning electron microscopy (SEM) and atomic force microscopy (AFM). SEM and AFM observations revealed that aggregated rGO was deposited on the GC electrode, forming a rather rough surface. The rGO-modified electrodes exhibited significantly higher responses in redox reactions of H_2_O_2_ as compared with the response of an unmodified GC electrode. In addition, the electrocatalytic activity of the rGO-modified electrode to hydrazine oxidation was also higher than that of the unmodified GC electrode. The response of the rGO-modified electrode was rationalized based on the higher catalytic activity of rGO to the redox reactions of H_2_O_2_ and hydrazine. The results suggest that rGO-modified electrodes are useful for constructing electrochemical sensors.

## 1. Introduction

A variety of functional materials have been utilized to modify the surface of electrodes for constructing electrochemical devices, which include catalysts [1,2], redox mediators [3,4], polymers [5,6,7,8], proteins [9,10,11], and DNA [12,13]. Recently, carbon nanomaterials have been widely employed for this purpose because of their high catalytic activity in redox reactions [14,15,16,17,18,19,20,21,22]. In this context, we have studied the catalytic activity of carbon nanotubes (CNTs) deposited on the surface of metal and carbon electrodes, and demonstrated high catalytic activity of CNTs in the oxidation of hydrogen peroxide (H_2_O_2_) [20]. CNT-modified electrodes have successfully been used to construct choline and lactate biosensors by combining the electrodes with enzymes [21,22]. In some cases, carbon CNTs have been combined with other functional materials to enhance catalytic activity [23,24].

Recently, graphene has attracted considerable attention from diverse fields in science and technology because of its excellent electrical and mechanical properties [25,26,27,28,29,30]. Graphene is a two-dimensional nanosheet consisting of sp^2^-hybridized carbon atoms arranged in a honeycomb structure. Graphene materials exhibit many advantages over CNTs, including higher surface area, structural simplicity, higher conductivity, and lower production cost. Thus, graphene materials have been applied to the development of optical and electrochemical sensors [31,32,33,34], energy storage [35,36], fuel cells [37], controlled release [38,39,40], and so forth. In the present study, we have prepared reduced grapheme oxide (rGO)-modified glassy carbon (GC) electrodes and have evaluated their catalytic activity with respect to redox reactions of H_2_O_2_ and hydrazine. rGO-modified electrodes were prepared by electrodeposition of graphene oxide (GO), in which GO was electrochemically reduced to form a thin layer of insoluble rGO on the surface of the GC electrode. The rGO-modified GC electrodes exhibited high catalytic activity to oxidation of H_2_O_2_ and hydrazine. We discuss the possible use of rGO-modified electrodes for constructing electrochemical sensors.

## 2. Experimental Section

### 2.1. Reagents

Graphite powder was obtained from Nakarai Co. (Kyoto, Japan). Hydrogen peroxide (30% aqueous solution) and hydrazine monohydrate were purchased from Santoku Chemical Co. (Tokyo, Japan) and Tokyo Kasei Co. (Tokyo, Japan), respectively. All other reagents were of the highest grade available and were used without further purification. We synthesized GO in accordance with the Hammers method [41].

### 2.2. Apparatus

We used atomic force microscopy (AFM, SPM-9600, Shimadzu Co., Kyoto, Japan) and scanning electron microscopy (SEM, S-3200N, Hitachi Co., Tokyo, Japan) for imaging GO and the surface of rGO-modified electrodes. All electrochemical measurements were performed using an electrochemical analyzer (Model 660B, ALS Co., Tokyo, Japan).

### 2.3. Deposition of rGO on the Surface of a GC Electrode

We deposited GO on the surface of a GC disk electrode (3-mm diameter) in accordance with a published procedure with slight modifications [42]. Briefly, the GC electrode was immersed in a dispersion of GO (0.3 mg/mL) in 0.7 mM pH 9 phosphate buffer. We then repeatedly scanned the electrode potential from −1.5 to 0.6 V (*vs.* Ag/AgCl electrode) at 10 mV s^−1^ with gentle stirring under a nitrogen atmosphere (~20 °C). The modified GC electrode thus prepared was rinsed thoroughly in working buffer before use.

### 2.4. AFM and SEM imaging

AFM images of GO were recorded in air at room temperature (~20 °C) using a SPM-9600 instrument operating in dynamic (tapping) mode. The sample for AFM observation was prepared on a mica surface. We obtained SEM images of the deposited rGO for platinum-sputtered samples (prepared on a GC plate) with an S-3200N instrument operating at 15 kV. AFM was also employed to study the surface morphology of the rGO-deposited GC plate.

### 2.5. Electrochemical Measurements

We measured the electrochemical response of rGO-modified and unmodified GC electrodes in a glass cell using the GC electrode as the working electrode, a platinum wire as the counter electrode, and a Ag/AgCl electrode (3.3 M KCl) as the reference electrode. All measurements were performed in air at room temperature (~20 °C).

## 3. Results and Discussion

### 3.1. Preparation of rGO-Modified GC Electrode

**Figure 1:**
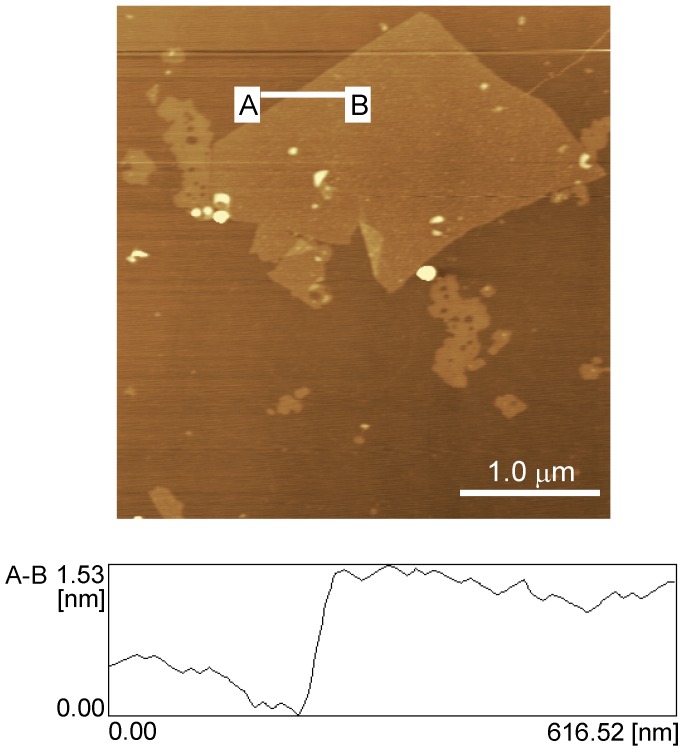
Atomic force microscopy (AFM) image of graphene oxide (GO) and its height profile.

Prior to depositing rGO on the surface of a GC electrode, we characterized the synthesized GO using AFM. Figure 1 shows a typical AFM image and height profile of a GO sheet deposited onto a mica substrate from an aqueous dispersion. The AFM image demonstrates that the GO sheet has a thickness of 1.0–1.5 nm and a length in the range of several micrometers. The observed thickness of the GO sheet agrees well with the reported average thickness of fully exfoliated GO sheets [43,44].

**Figure 2:**
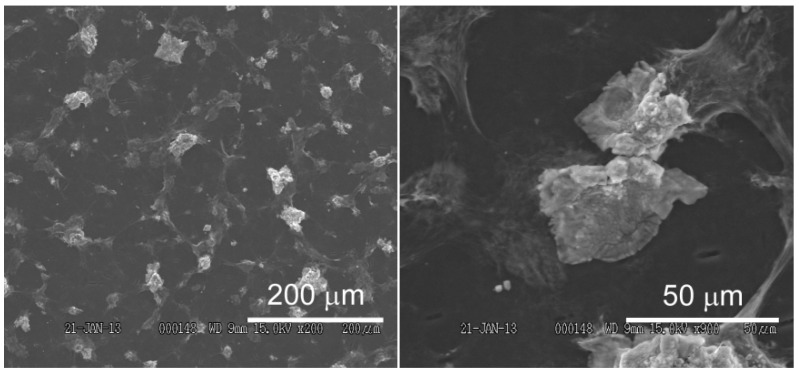
Scanning electron microscopy (SEM) images of the surface of an rGO-modified glassy carbon (GC) electrode.

**Figure 3:**
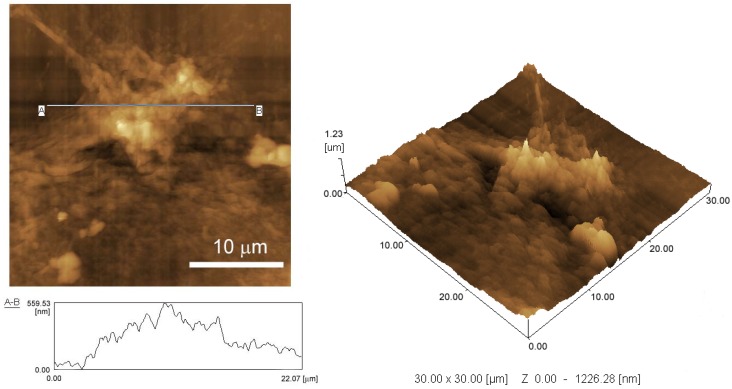
AFM images of rGO aggregates deposited on a GC electrode and its height profile.

We electrochemically deposited the GO sheets on the surface of a GC electrode. GO is converted to rGO during electrodeposition [42]. The surface morphology of the modified GC electrode was studied by SEM and AFM. Figure 2 shows SEM images of the surface of an rGO-modified GC electrode. The surface of the GC electrode was covered with an rGO layer containing large aggregates 10–30 μm in length (Figure 2B). Figure 3 shows AFM images and a height profile of the rGO aggregate, demonstrating that the rGO aggregate has a height of >500 nm. The SEM and AFM images clearly show that the surface of an rGO-modified GC electrode is rather rough, which is attributable to rGO aggregate formation. It is likely that, during electrodeposition, GO was electrochemically reduced to form highly hydrophobic rGO when the electrode potential was negatively scanned, which resulted in deposition of aggregated rGO on the electrode surface.

### 3.2. Redox Reactions of H_2_O_2_ and Hydrazine on an rGO-Modified Electrode

**Figure 4:**
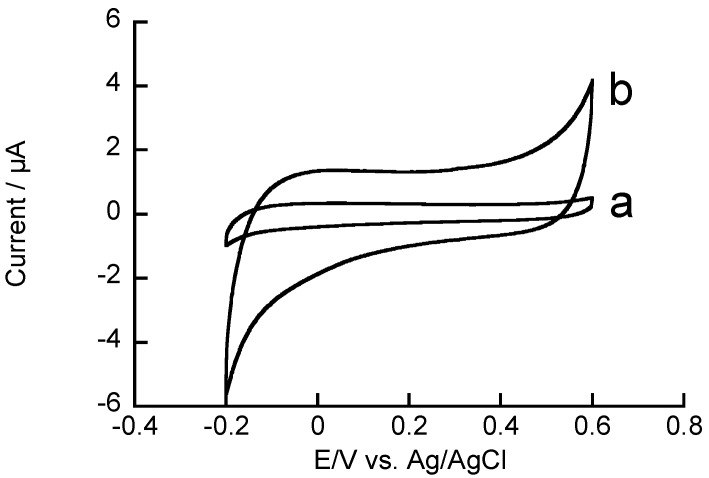
Cyclic voltammograms (CVs) of 3 mM H_2_O_2_ on (**a**) unmodified GC and (**b**) rGO-modified electrodes in 0.1 M pH 7.4 phosphate buffer. Scan rate: 0.1 V·s^−1^.

The rGO-modified electrode may exhibit high catalytic activity in redox reactions because a large quantity of rGO was deposited on the GC electrode. Edge-plane-like defective sites on rGO facilitate electron transfer to molecules in solution [45]. To evaluate the catalytic activity of rGO-modified GC electrodes, we recorded cyclic voltammograms (CVs) of H_2_O_2_ (Figure 4). The CV of H_2_O_2_ on an rGO-modified GC electrode exhibited oxidation and reduction currents, the onset potentials of which were 0.4 and 0.1 V, respectively, whereas the unmodified GC electrode exhibited virtually no redox response to H_2_O_2_ in the potential range tested. Thus, the rGO-modified GC electrode exhibited a significantly higher response to H_2_O_2_ than the unmodified electrode. These results suggest that an rGO-modified electrode can be used for amperometric quantitation of H_2_O_2_.

**Figure 5:**
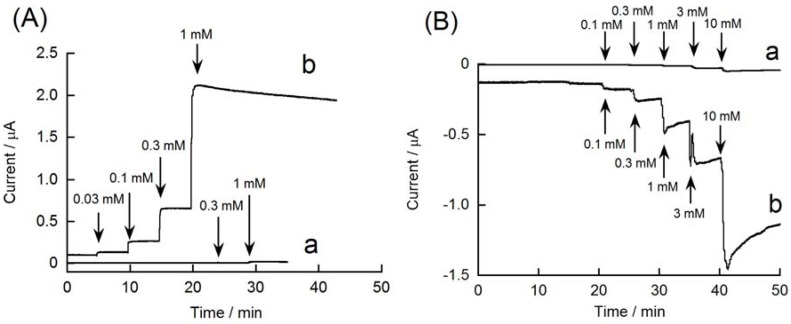
Amperometric responses of (**a**) unmodified and (**b**) rGO-modified GC electrodes to H_2_O_2_, recorded at (**A**) 0.6 V and (**B**) −0.1 V.

Figure 5A depicts the amperometric response of rGO-modified and unmodified GC electrodes at 0.6 V. The oxidation current of H_2_O_2_ increased with increasing H_2_O_2_ concentration, whereas the response of the unmodified GC electrode was negligible. The rGO-modified electrode can also be operated at −0.1 V to detect a reduction current of H_2_O_2_ (Figure 5B). The reduction current increased in accordance with the concentration of H_2_O_2_. Yang and coworkers have recently reported that GC electrodes modified with GO-Ag composite exhibit amperometric response to 0.0016–9.0 mM H_2_O_2_ [46]. These results suggest that an rGO-modified electrode can be used for constructing biosensors by combining oxidase enzymes, such as glucose oxidase, lactate oxidase, and cholesterol oxidase, because these enzymes generate H_2_O_2_ as reaction products.

**Figure 6:**
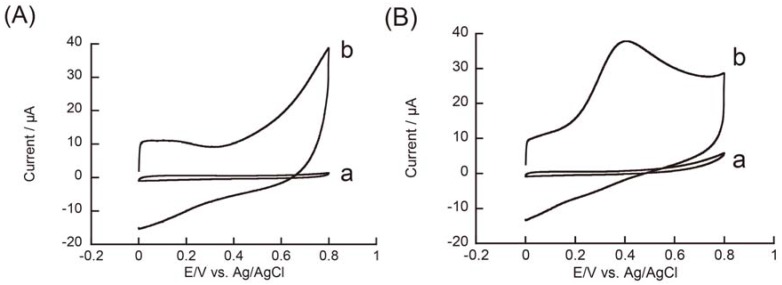

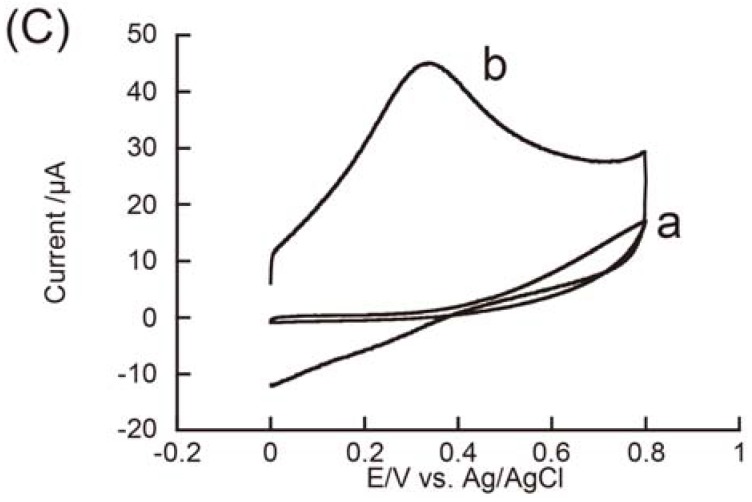
CVs of 3 mM hydrazine recorded on (**a**) unmodified GC and (**b**) rGO-modified electrodes at (**A**) pH 5.0; (**B**) 7.0; and (**C**) 9.0.

We also studied the redox reaction of hydrazine on an rGO-modified electrode. Figure 6 shows CVs of hydrazine recorded on rGO-modified and unmodified GC electrodes at pH 5.0, 7.0, and 9.0. The unmodified GC electrode exhibited no redox peak over the potential range of 0–0.8 V under the present experimental conditions, although the oxidation current slightly increased at 0.6–0.8 V in pH 9.0 medium. In contrast, we observed well-defined oxidation peaks in CVs at 0.35–0.4 V in pH 7.0 and 9.0 solutions on an rGO-modified electrode. No reduction peak was observed in the CVs during the reverse scan, demonstrating that the redox process was irreversible. We did not observe an oxidation peak at pH 5.0 in the potential range of 0–0.8 V, although the oxidation current gradually increased at 0.4–0.8 V. The oxidation current in CVs recorded at pH 7.0 and 9.0 was remarkably high. In this context, Wang and coworkers also reported an enhanced redox response of hydrazine on GC electrodes modified with polymer-coated graphene [47]. Electrocatalytic oxidation of hydrazine was pH-dependent, that is, higher in neutral or basic solutions [48], in accordance with the pH-dependent response of hydrazine on the rGO-modified electrode.

**Figure 7:**
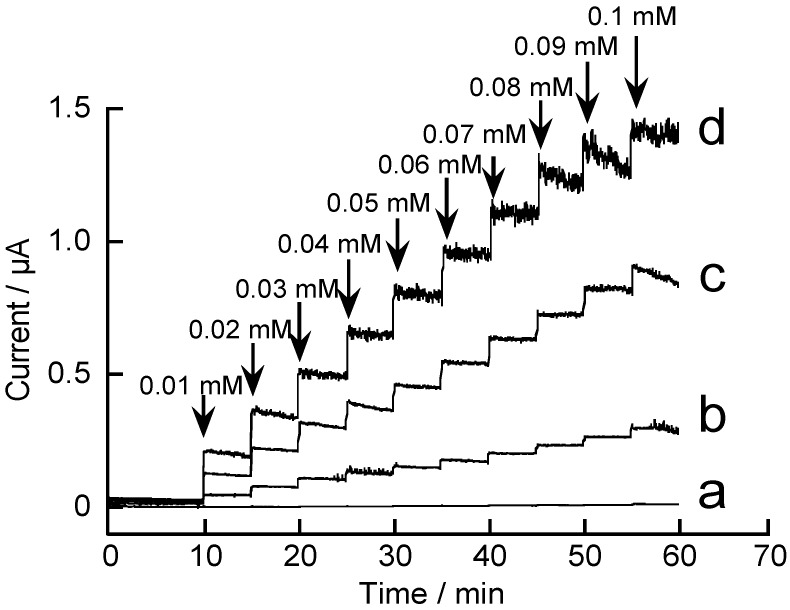
Amperometric responses of (**a**) unmodified and (**b**–**d**) rGO-modified electrodes to hydrazine, recorded at 0.4 V in 0.1 M pH 7.0 phosphate buffer. The rGO-modified electrodes were prepared by scanning electrode potential (**b**) six; (**c**) seven; and (**d**) eight times.

It is interesting to evaluate the amperometric response of rGO-modified GC electrodes to hydrazine. Figure 7 shows the amperometric response of the rGO-modified GC electrodes to 0.01–0.1 mM hydrazine in solution at pH 7.0.

**Figure 8:**
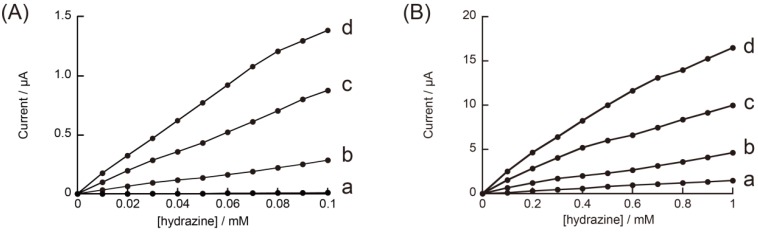
Calibration graphs of (**a**) unmodified and (**b**–**d**) rGO-modified electrodes to hydrazine, recorded at 0.4 V in 0.1 M pH 7.0 phosphate buffer. Hydrazine concentration: (**A**) 0.01–0.1 mM; (**B**) 0.1–1 mM.

In this experiment, we used three types of rGO-modified electrodes, in which the quantity of rGO loaded on the electrode surface was regulated by changing the number of scans of electrode potential upon electrode position of rGO. The quantity of the deposited rGO on an electrode depends on the number of scans during electrodeposition [42]. Higher quantities of rGO can be deposited on the electrode surface by increasing the number of potential scans. Thus, we fabricated three different types of rGO-modified GC electrodes by changing the number of potential scans (*i.e.*, six, seven, and eight) during electrodeposition. The rGO-modified electrode prepared by scanning the electrode potential eight times exhibited the highest response among the modified electrodes tested. The response of the modified electrode prepared by scanning six times was significantly lower than that of the other electrodes, suggesting that scanning the electrode potential seven or eight times is required for depositing an adequate quantity of rGO on the electrode surface. The results show that rGO-modified electrodes are sensitive to hydrazine in the concentration range of 0.01–0.1 mM. In a separate measurement, we have confirmed that the modified electrodes also exhibit an amperometric response to hydrazine in a higher concentration range, 0.1–1.0 mM (data not shown). Figure 8 shows calibration graphs of the electrodes to hydrazine in the concentration range of 0.01–0.1 and 0.1–1.0 mM. The results suggest a possible use of rGO-modified GC electrodes for quantitating hydrazine at submillimolar concentrations. Recently, electrochemical oxidation of hydrazine on GC electrodes modified with metal nanoparticle-rGO composite has been reported [49].

## 4. Conclusions

We have demonstrated that rGO-modified GC electrodes can be prepared by electrodeposition of GO by scanning the electrode potential. The rGO-modified GC electrodes exhibited high sensitivity in voltammetric and amperometric measurements of H_2_O_2_ and hydrazine. The sensitivity of the modified electrodes can be tuned by regulating the loading of rGO on the electrode surface. Therefore, the rGO-modified GC electrodes are promising with respect to electrochemical sensor development. Further research for improving the performance characteristics of rGO-modified GC electrodes is now in progress in our laboratory.
